# Supplementary material for: Relative Efficacy and Safety of Anti-Inflammatory Biologic Agents for Osteoarthritis: A Conventional and Network Meta-Analysis
Source: J Clin Med. 2022 Jul 7;11(14):3958. doi: 10.3390/jcm11143958 (PMC9317938; doi:10.3390/jcm11143958)
Supplement: Supplementary file 1 [file jcm-11-03958-s001.zip › jcm-1737765-supplementary.pdf]

Supplementary Materials

Table S1. PRISMA extension statement for network meta-analysis.
Table S2. Literature search procedures and strategies.
Table S3. A hierarchy list of data extraction.
Table S4. Rank probability of pain.
Table S5. Rank probability of physical function.
Table S6. Network meta-analysis of stiffness for different interventions.
Table S7. Rank probability of stiffness.
Table S8. Network meta-analysis of AEs for different interventions.
Table S9. Sensitivity analysis in pain, stiffness and function.
Table S10. Sensitivity analysis in adverse events.
Table S11. Node-splitting analyses of pain.
Table S12. ISD of inconsistency test.
Table S13. Results of Egger's test.

Figure S1. Results of the conventional meta-analysis of pain.
Figure S2. Results of the conventional meta-analysis of physical function.
Figure S3. Results of the conventional meta-analysis of stiffness.
Figure S4. Results of the conventional meta-analysis of adverse events.
Figure S5. Publication bias examined by funnel plot.

Table S1: PRISMA extension statement for network meta-analysis

Section/Topic	Item #	Checklist Item	Reported on Page #
**TITLE**	1	Identify the report as a systematic review incorporating a network meta-analysis (or related form of meta-analysis)	1
Title			
**ABSTRACT**			
Structured summary	2	Provide a structured summary including, as applicable:<br>**Background**: main objectives<br>**Methods**: data sources; study eligibility criteria, participants, and interventions; study and synthesis methods, such as network meta-analysis.<br>**Results**: number of studies and participants identified; summary estimates with corresponding confidence/credible intervals; treatment rankings may also be	2,3

		discussed. Authors may choose to summarize pairwise comparisons against a chosen treatment included in their analyses for brevity.<br>**Discussion/Conclusion**: limitations; conclusions and implications of findings.<br>**Other**: primary source of funding; systematic review registration number with registry name	
**INTRODUCTION**			
Rationale	3	Describe the rationale for the review in the context of what is already known, including mention of why a network meta-analysis has been conducted.	4
Objectives	4	Provide an explicit statement of questions being addressed, with reference to participants, interventions, comparisons, outcomes, and study design (PICOS).	4,5
**METHODS**			
Protocol and registration	5	Indicate whether a review protocol exists and if and where it can be accessed (e.g., Web address); and, if available, provide registration information, including registration number.	5
Eligible criteria	6	Specify study characteristics (e.g., PICOS, length of follow-up) and report characteristics (e.g., years considered, language, publication status) used as criteria for eligibility, giving rationale. Clearly describe eligible treatments included in the treatment network, and note whether any have been clustered or merged into the same node (with justification).	5,6
Information sources	7	Describe all information sources (e.g., databases with dates of coverage, contact with study authors to identify additional studies) in the search and date last searched.	5
Search	8	Present full electronic search strategy for at least one database, including any limits used, such that it could be repeated.	Online resource Appendix. 1
Study selection	9	State the process for selecting studies (i.e., screening, eligibility, included in systematic review, and, if applicable, included in the meta-analysis).	6
Data collection process	10	Describe method of data extraction from reports (e.g., piloted forms, independently, in duplicate) and any processes for obtaining and confirming data from investigators.	6,7
Data items	11	List and define all variables for which data were sought (e.g., PICOS, funding sources) and any assumptions and simplifications made.	6
**Geometry of the network**	**S1**	Describe methods used to explore the geometry of the treatment network under study and potential biases related to it. This should include how the evidence base has been graphically summarized for presentation, and what characteristics were compiled and used to describe the evidence bae to readers.	9,10
Risk of bias within individual studies	12	Describe methods used for assessing risk of bias of individual studies (including specification of whether this was done at the study or outcome level), and how this information is to be used in any data synthesis.	7,8
Summary measures	13	State the principal summary measures (e.g., risk ratio, difference in means). Also describe the use of additional summary measures assessed, such as treatment rankings and surface under the cumulative ranking curve (SUCRA) values, as well as modified approaches used to present summary findings from meta-analyses.	8,9

Planned methods of analysis	14	Describe the methods of handling data and combining results of studies for each network meta-analysis. This should include, but not be limited to:<br>• Handling of multi-arm trials;<br>• Selection of variance structure;<br>• Selection of prior distributions in Bayesian analyses; and<br>• Assessment of model fit.	8,9
**Assessment of Inconsistency**	**S2**	Describe the statistical methods used to evaluate the agreement of direct and indirect evidence in the treatment network(s) studied. Describe efforts taken to address its presence when found.	8,9
Risk of bias across studies	15	Specify any assessment of risk of bias that may affect the cumulative evidence (e.g., publication bias, selective reporting within studies).	7,8
Additional analyses	16	Describe methods of additional analyses if done, indicating which were pre-specified. This may include, but not be limited to, the following:<br>• Sensitivity or subgroup analyses;<br>• Meta-regression analyses;<br>• Alternative formulations of the treatment network; and<br>• Use of alternative prior distributions for Bayesian analyses (if applicable).	8
**RESULTS**			
Study selection	17	Given numbers of studies screened, assessed for eligibility, and included in the review, with reasons for exclusions at each stage, ideally with a flow diagram.	9
Presentation of network structure	S3	Provide a network graph of the included studies to enable visualization of the geometry of the treatment network.	22 (Fig. 2)
Summary of network geometry	S4	Provide a brief overview of characteristics of the treatment network. This may include commentary on the abundance of trials and randomized patients for different interventions and pairwise comparisons in the network, gaps of evidence in the treatment network, and potential biases reflected by the network structure.	9,10
Study characteristics	18	For each study, present characteristics for which data was extracted (e.g., study size, PICOS, follow-up period) and provide the citations.	9,10,23,24
Risk of bias within studies	19	Present data on risk of bias of each study and, if available, any outcome level assessment.	12,13
Results of individual studies	20	For all outcomes considered (benefits or harms), present, for each study: 1) simple summary data for each intervention group, and 2) effect estimates and confidence intervals. Modified approaches may be needed to deal with information from larger networks.	10,11,12
Synthesis of results	21	Present results of each meta-analysis done, including confidence/credible intervals. In larger networks, authors may focus on comparisons versus a particular comparator (e.g. placebo or standard care), with full findings presented in an appendix. League tables and forest plots may be considered to summarize pairwise comparisons. If additional summary measures were explored (such as treatment rankings), these should also be presented.	10,11,12
Exploration for inconsistency	S5	Describe results from investigations of inconsistency. This may include such information	12

		as measures of model fit to compare consistency and inconsistency models, *P* values from statistical tests, or summary of inconsistency estimates from different parts of the treatment network.	
Risk of bias across studies	22	Present results of any assessment of risk of bias across studies for the evidence base being studied.	12,13
Results of additional analyses	23	Give results of additional analyses, if done (e.g., sensitivity or subgroup analyses, meta-regression analyses, alternative network geometries studied, alternative choice of prior distributions for Bayesian analyses, and so forth).	13
**DISCUSSION**			
Summary of evidence	24	Summarize the main findings, including the strength of evidence for each main outcomes; consider their relevance to key groups (e.g., healthcare providers, users, and policy-makers).	13
Limitations	25	Discuss limitations at study and outcome level (e.g., risk of bias), and review level (e.g., incomplete retrieval of identified research, reporting bias). Comment on the validity of the assumptions, such as transitivity and consistency. Comment on any concerns regarding network geometry (e.g., avoidance of certain comparisons).	16
Conclusion	26	Provide a general interpretation of the results in the contest of other evidence, and implications for future research.	17
FUNDING			
Funding	27	Describe sources of funding for the systematic review and other support (e.g., supple of data); role of funders for the systematic review. This should also include information regarding whether funding has been received from manufacturers of treatment in the network and/or whether some of the authors are content experts with professional conflicts of interest that could affect use of treatments in the network.	17

Table S2. Literature search procedures and strategies.

PubMed

No.	Query	Results
1	"osteoarthritis"[MeSH Terms] OR "osteoarthritis"[Title/Abstract] OR "Osteoarthritides"[Title/Abstract] OR "Osteoarthrosis"[Title/Abstract] OR "Osteoarthroses"[Title/Abstract] OR "Arthrosis"[Title/Abstract] OR "Arthroses"[Title/Abstract] OR "Degenerative Arthritides"[Title/Abstract] OR "Degenerative Arthritis"[Title/ Abstract] OR "Osteoarthrosis Deformans"[Title/Abstract]	96,093

**2**	"Tumor Necrosis Factor Inhibitors"[Mesh] OR "Tumor Necrosis Factor Inhibitors"[Title/Abstract] OR "Tumor Necrosis Factor Inhibitor"[Title/Abstract] OR "TNF Antagonists"[Title/Abstract] OR "TNF Antagonist"[Title/Abstract] OR "TNF Inhibitors"[Title/Abstract] OR "TNF Inhibitor"[Title/Abstract] OR "TNF Blockers"[Title/Abstract] OR "TNF Blocker"[Title/Abstract] OR "Tumor Necrosis Factor Antagonists"[Title/Abstract] OR "Tumor Necrosis Factor Antagonist"[Title/Abstract] OR "Tumor Necrosis Factor Blockers"[Title/Abstract] OR "Tumor Necrosis Factor Blocker"[Title/Abstract] OR "Tumor Necrosis Factor-alpha Antagonists"[Title/Abstract] OR "Tumor Necrosis Factor-alpha Antagonist"[Title/Abstract] OR "Tumor Necrosis Factor-alpha Inhibitors"[Title/Abstract] OR "Tumor Necrosis Factor-alpha Inhibitor"[Title/Abstract] OR "Tumor Necrosis Factor-alpha Blockers"[Title/Abstract] OR "Tumor Necrosis Factor-alpha Blocker"[Title/Abstract]	5,760
**3**	Adalimumab[Mesh] OR Humira[Title/Abstract] OR Adalimumab[Title/Abstract] OR Amjevita[Title/Abstract] OR Cyltezo[Title/Abstract] OR "Adalimumab-adbm"[Title/Abstract] OR "Adalimumab-atto"[Title/Abstract]	8,674
**4**	Infliximab[Mesh] OR Infliximab[Title/Abstract] OR Renflexis[Title/Abstract] OR Inflectra[Title/Abstract] OR Remicade[Title/Abstract] OR "Monoclonal Antibody cA2"[Title/Abstract] OR "MAb cA2"[Title/Abstract] OR "Infliximab-abda"[Title/Abstract] OR "Infliximab-dyyb"[Title/Abstract]	15,177
**5**	Etanercept [MeSH] OR Etanercept [Title/Abstract] OR "TNFR-Fc Fusion Protein" [Title/Abstract] OR "TNFR Fc Fusion Protein" [Title/Abstract] OR "TNR 001" [Title/Abstract] OR "TNT Receptor Fusion Protein" [Title/Abstract] OR "TNTR-Fc" [Title/Abstract] OR "TNR-001" [Title/Abstract] OR "TNR001" [Title/Abstract] OR "Etanercept-szzs" [Title/Abstract] OR "TNF Receptor Type II-IgG Fusion Protein" [Title/Abstract] OR "TNF Receptor Type II IgG Fusion Protein" [Title/Abstract] OR "Erelzi" [Title/Abstract] OR "Enbrel" [Title/Abstract] OR "Recombinant Human Dimeric TNF Receptor Type II-IgG Fusion Protein" [Title/Abstract] OR	8,743

	"Recombinant Human Dimeric TNF Receptor Type II IgG Fusion Protein" [Title/Abstract]	
6	"Certolizumab Pegol" [MeSH] OR "Certolizumab Pegol"[Title/Abstract] OR Cimzia [Title/Abstract] OR CDP870 [Title/Abstract] OR "CDP 870" [Title/Abstract]	1,052
7	golimumab [Supplementary Concept] OR "CNTO-148"[Title/Abstract] OR "CNTO 148" [Title/Abstract] OR Simponi [Title/Abstract]	649
8	#2 OR #3 OR #4 OR #5 OR #6 OR #7	27,571
9	AMG 108 [Supplementary Concept] OR "AMG-108"[Title/Abstract] OR AMG108[Title/Abstract]	4
10	"Canakinumab"[Supplementary Concept] OR "Canakinumab"[Title/Abstract] OR "ilaris"[Title/Abstract] OR "ACZ-885"[Title/Abstract] OR "ACZ885"[Title/Abstract]	749
11	"lutikizumab" [Supplementary Concept] OR ABT-981[Title/Abstract] OR lutikizumab[Title/Abstract]	6
12	"Interleukin 1 Receptor Antagonist Protein"[Mesh] OR "Interleukin 1 Receptor Antagonist Protein"[Title/ Abstract] OR Anakinra[Title/Abstract] OR IL-1Ra[Title/Abstract] OR "Urine IL-1 Inhibitor"[Title/Abstract] OR Antril[Title/Abstract] OR Kineret[Title/Abstract] OR "Urine Derived IL1 Inhibitor"[Title/Abstract] OR "Urine-Derived IL1 Inhibitor"[Title/Abstract] OR "IL1 Febrile Inhibitor"[Title/Abstract]	9,108
13	gevokizumab[Supplementary Concept] OR gevokizumab[Title/Abstract] OR "XOMA 052"[Title/Abstract] OR XOMA052[Title/Abstract] OR XOMA-052[Title/Abstract] OR "XMA005.2"[Title/Abstract] OR "XMA 005.2"[Title/ Abstract] OR "XMA-005.2"[Title/Abstract]	53

**14**	"antagonists and inhibitors"[MeSH Subheading] OR "inhibitor"[Title/Abstract] OR "antagonist"[Title/Abstract] OR "blocker"[Title/Abstract] OR "inhibitors"[Title/Abstract] OR "antagonists"[Title/Abstract] OR "blockers"[Title/Abstract] OR "antagonists and inhibitors"[Title/Abstract]	1,564,306
**15**	"interleukin-17"[MeSH Terms] OR "interleukin-17"[Title/Abstract] OR "CTLA-8"[Title/Abstract] OR "interleukin-17"[Title/Abstract] OR "IL-17"[Title/Abstract] OR "interleukin 17f"[Title/Abstract] OR "interleukin 17f"[Title/Abstract] OR "IL-17F"[Title/Abstract] OR "cytokine ml 1"[Title/Abstract] OR "cytokine ml 1"[Title/Abstract] OR "interleukin 17c"[Title/Abstract] OR "interleukin 17c"[Title/Abstract] OR "IL-17C"[Title/Abstract] OR "interleukin 17e"[Title/Abstract] OR "interleukin 17e"[Title/Abstract] OR "interleukin 25"[Title/Abstract] OR "interleukin 25"[Title/Abstract] OR "il 17e"[Title/Abstract] OR "il 17e"[Title/Abstract] OR "interleukin 17a"[Title/Abstract] OR "interleukin 17a"[Title/Abstract] OR "cytotoxic t lymphocyte associated antigen 8"[Title/Abstract] OR "cytotoxic t lymphocyte associated antigen 8"[Title/Abstract] OR "IL-17A"[Title/Abstract] OR "CTLA8"[Title/Abstract] OR "interleukin 17b"[Title/Abstract] OR "interleukin 17b"[Title/Abstract] OR "IL-17B"[Title/Abstract]	25,224
**16**	"interleukin-6"[MeSH Terms] OR "interleukin-6"[Title/Abstract] OR "interleukin 6"[Title/Abstract] OR "IL6"[Title/ Abstract] OR "IL-6"[Title/Abstract] OR "b cell stimulatory factor 2"[Title/Abstract] OR "b cell stimulatory factor-2"[Title/Abstract] OR "BSF-2"[Title/Abstract] OR "b cell differentiation factor 2"[Title/Abstract] OR "b cell differentiation factor-2"[Title/Abstract] OR "Hybridoma Growth Factor"[Title/Abstract] OR "IFN-beta 2"[Title/Abstract] OR "Plasmacytoma Growth Factor"[Title/Abstract] OR "MGI-2"[Title/Abstract] OR "hepatocyte stimulating factor"[Title/Abstract] OR "hepatocyte-stimulating factor"[Title/Abstract] OR "myeloid differentiation-inducing protein"[Title/Abstract] OR "myeloid differentiation inducing protein"[Title/Abstract] OR "b-cell	149,168

	differentiation factor"[Title/Abstract] OR "b cell differentiation factor"[Title/Abstract] OR "interferon beta 2"[Title/Abstract] OR "interferon beta-2"[Title/Abstract] OR "b cell stimulatory factor 2"[Title/Abstract] OR "b cell stimulatory factor-2"[Title/Abstract]	
**17**	"interleukins"[MeSH Terms] OR "interleukin"[Title/Abstract] OR "interleukin"[Title/Abstract]	341,768
**18**	(#15 OR #16 OR #17) AND #14	70,339
**19**	tocilizumab[Supplementary Concept] OR tocilizumab[Title/Abstract] OR RHPM-1[Title/Abstract] OR RG-1569[Title/Abstract] OR R-1569[Title/Abstract] OR MSB11456[Title/Abstract] OR MSB-11456[Title/ Abstract] OR atlizumab[Title/Abstract] OR RO-4877533[Title/Abstract] OR Actemra[Title/Abstract] OR roactemra[Title/Abstract]	3,759
**20**	sarilumab[Supplementary Concept] OR sarilumab[Title/Abstract] OR SAR-153191[Title/Abstract] OR SAR153191[Title/Abstract] OR Kevzara[Title/Abstract] OR REGN-88[Title/Abstract] OR REGN88[Title/Abstract]	157
**21**	ALX-0061[Supplementary Concept] OR ALX-0061[Title/Abstract] OR vobarilizumab [Title/Abstract]	4
**22**	SANT-7[Supplementary Concept] OR SANT-7[Title/Abstract] OR SANT7[Title/Abstract]	18
**23**	satralizumab[Supplementary Concept] OR satralizumab[Title/Abstract]	17
**24**	sirukumab[Supplementary Concept] OR sirukumab[Title/Abstract] CNTO-136[Title/Abstract] OR "CNTO 136"[Title/Abstract]	3
**25**	siltuximab[Supplementary Concept] OR siltuximab[Title/Abstract] OR CLLB8[Title/Abstract] OR "cClB8 monoclonal antibody"[Title/Abstract] OR Sylvant[Title/Abstract] OR CNTO-	171

	328[Title/Abstract] OR "monoclonal antibody CNTO-328"[Title/Abstract] OR "CNTO 328 monoclonal antibody"[Title/Abstract] OR "monoclonal antibody CNTO 328"[Title/Abstract] OR "monoclonal antibody CNTO328"[Title/Abstract]	
**26**	clazakizumab[Supplementary Concept] OR clazakizumab[Title/Abstract] OR ALD-518[Title/Abstract] OR ALD518[Title/Abstract] OR BMS-945429[Title/Abstract]	25
**27**	olokizumab[Supplementary Concept] OR olokizumab[Title/Abstract] OR CDP-6038[Title/Abstract] OR CDP6038[Title/Abstract]	16
**28**	Secukinumab[Supplementary Concept] OR Secukinumab[Title/Abstract] OR Cosentyx[Title/Abstract] OR "AIN 457"[Title/Abstract] OR AIN457[Title/Abstract] OR AIN-457[Title/Abstract]	1,177
**29**	Ixekizumab[Supplementary Concept] OR Ixekizumab[Title/Abstract] OR Taltz[Title/Abstract] OR LY2439821[Title/Abstract] OR LY-2439821[Title/Abstract]	587
**30**	Brodalumab[Supplementary Concept] OR Brodalumab[Title/Abstract] OR Siliq[Title/Abstract] OR KHK-4827[Title/Abstract] OR KHK4827[Title/Abstract] OR AMG-827[Title/Abstract] OR "AMG 827"[Title/Abstract]	337
**31**	#9 OR #10 OR #11 OR #12 OR #13 OR #18 OR #19 OR #20 OR #21 OR #22 OR #23 OR #24 OR #25 OR #26 OR #27 OR #28 OR #29 OR #30	77,779
**32**	"randomized controlled trial"[Publication Type] OR "controlled clinical trial"[Publication Type] OR "randomized"[Title/Abstract] OR "randomised"[Title/Abstract] OR "randomization"[Title/Abstract] OR "randomisation"[Title/Abstract] OR "randomly"[Title/Abstract] OR "placebo"[Title/Abstract] OR "trial"[Title/Abstract]	1,433,625
**33**	"observational study"[publication type]	86,304
**34**	(#8 OR #31) AND #1 AND (#32 OR #33)	89

**35**	(#8 OR #31) AND #1 AND #32	87

Web of Science

No.	Query	Results
**1**	TS=(osteoarthritis OR Osteoarthritides OR Osteoarthrosis OR Osteoarthroses OR Arthrosis OR Arthroses OR "Degenerative Arthritides" OR "Degenerative Arthritis" OR "Osteoarthrosis Deformans")	136,805
**2**	TS=("Tumor Necrosis Factor Inhibitors" OR "TNF Antagonists" OR "TNF Inhibitors" OR "TNF Blockers" OR "T umor Necrosis Factor Antagonists" OR "Tumor Necrosis Factor Blockers" OR "Tumor Necrosis Factor-a (TNFa) Antagonists" OR "Tumor Necrosis Factor-a (TNF-a) Inhibitors" OR "Tumor Necrosis Factor-a (TNFa) Blockers")	5,161
**3**	TS=(Adalimumab OR Humira OR Adalimumab-adbm OR Amjevita OR Adalimumabatto OR Cyltezo OR "D2E7 Antibody")	18,836
**4**	TS=(Infliximab OR "Monoclonal Antibody cA2" OR "MAb cA2" OR Inflixímababda OR Renflexis OR Infliximab-dyyb OR Inflectra OR Remicade)	32,615
**5**	TS=(Etanercept OR "TNFR-Fc Fusion Protein" OR "TNFR Fc Fusion Protein" OR "TNR 001" OR "TNT Receptor Fusion Protein" OR "TNT R-Fc" OR "TNR-001" OR "TNR001" OR "Etanercept-szzs" OR "TNF Receptor Type II-IgG Fusion Protein" OR "TNF Receptor Type II IgG Fusion Protein" OR "Erelzi" OR "Enbrel" OR "Recombinant Human Dimeric TNF Receptor Type II-IgG Fusion Protein" OR "Recombinant Human Dimeric TNF Receptor Type II IgG Fusion Protein")	17,522
**6**	TS=("Certolizumab Pegol" OR "Certolizumab Pegol" OR Cimzia OR CDP870 OR "CDP 870")	2,702

**7**	TS=(golimumab OR “CNTO-148” OR “CNTO 148” OR Simponi)	3,173
**8**	#2 OR #3 OR #4 OR #5 OR #6 OR #7	53,983
**9**	TS=(“AMG 108” OR AMG108 OR AMG-108)	8
**10**	TS=(Canakinumab OR ilaris OR ACZ-885 OR ACZ885)	1,413
**11**	TS=(lutikizumab OR ABT-981)	21
**12**	TS=(“Interleukin 1 Receptor Antagonist Protein” OR Anakinra OR IL-1Ra OR “Urine IL-1 Inhibitor” OR Antril O R Kineret OR "Urine Derived IL1 Inhibitor" OR "Urine-Derived IL1 Inhibitor" OR "IL1 Febrile Inhibitor")	12,104
**13**	TS=(gevokizumab OR “XOMA 052” OR XOMA052 OR XOMA-052 OR “XMA005.2” OR “XMA 005.2” OR “XMA -005.2”)	102
**14**	TS=("antagonists and inhibitors" OR "inhibitor" OR "antagonist" OR "blocker" OR "inhibitors" OR "antagonist s" OR "blockers")	2,626,051
**15**	TS=(interleukin-17 OR “Interleukin 17” OR CTLA-8 OR IL-17 OR Interleukin-17F OR “Interleukin 17F” OR IL-1 7F OR “Cytokine ML-1” OR “Cytokine ML 1” OR Interleukin-17C OR “Interleukin 17C” OR IL-17C OR “Cytoki ne CX2” OR Interleukin-17E OR “Interleukin 17E” OR Interleukin-25 OR “Interleukin 25” OR IL-17E OR “IL 17E ” OR Interleukin-17A OR “Interleukin 17A” OR “Cytotoxic T lymphocyte- Associated Antigen 8” OR “Cytotoxic T lymphocyte Associated Antigen 8” OR IL-17A OR CTLA8 OR Interleuk in-17B OR “Interleukin 17B” OR IL-17B)	39,603

**16**	TS=(interleukin-6 OR “interleukin 6” OR IL6 OR “B-Cell Stim-ulatory Factor 2” OR “B-Cell Stimulatory Factor-2” OR “B-Cell Differentiation Factor-2” OR “B-Cell Differentiation Factor 2” OR BSF-2 OR “Hybridoma Growth Fac-tor” OR “IFNbeta 2” OR “Plasmacy-toma Growth Factor” OR “Hepato-cyte-Stimulating Factor” OR “Hepatocyte Stimulating Fac-tor” OR MGI-2 OR “Myeloid DifferentiationInducing Protein” OR “Myeloid Differentiation Inducing Protein” OR “B-Cell Differentiation Factor” OR “B Cell Differentiation Factor” OR IL-6 OR “Interferon beta-2” OR “Interferon be ta 2” OR “B Cell Stimulatory Factor-2” OR “B Cell Stimulatory Factor 2”)	213,294
**17**	TS=(interleukins OR interleukin)	552,883
**18**	(#15 OR #16 OR #17) AND #14	119,490
**19**	TS=(tocilizumab OR RHPM-1 OR RG-1569 OR R-1569 OR MSB11456 OR MSB-11456 OR atlizumab OR RO4877533 OR Actemra OR roactemra)	7,761
**20**	TS=(sarilumab OR SAR-153191 OR SAR153191 OR Kev-zara OR REGN-88 OR REGN88)	394
**21**	TS=(ALX-0061 OR vobarilizumab)	20
**22**	TS=(SANT-7 OR SANT7)	37
**23**	TS=(satralizumab)	45
**24**	TS=(siltuximab OR CLLB8 OR “cClB8 monoclonal antibody” OR Sylvant OR CNTO-328 OR “monoclonal anti body CNTO-328” OR “CNTO 328 monoclonal antibody” OR “monoclonal antibody CNTO 328” OR “monoclo nal antibody CNTO328”)	352
**25**	TS=(sirukumab OR CNTO-136 OR “CNTO 136”)	135

**26**	TS=(clazakizumab OR ALD-518 OR ALD518 OR BMS-945429)	66
**27**	TS=(olokizumab OR CDP-6038 OR CDP6038)	35
**28**	TS=(Secukinumab OR Cosentyx OR “AIN 457” OR AIN457 OR AIN-457)	2,469
**29**	TS=(Ixekizumab OR Taltz OR LY2439821 OR LY-2439821)	1,075
**30**	TS=(Brodalumab OR Siliq OR KHK-4827 OR KHK4827 OR AMG-827 OR “AMG 827”)	514
**31**	#9 OR #10 OR #11 OR #12 OR #13 OR #18 OR #19 OR #20 OR #21 OR #22 OR #23 OR #24 OR #25 OR #26 OR #27 OR #28 OR #29 OR #30	132,395
**32**	TS=(Randomized controlled trial)	577,604
**33**	TS=(observational study)	227,380
**34**	(#8 OR #31) AND #1 AND (#32 OR #33)	102

Embase

No.	Query	Results
**1**	('osteoarthritis'/exp OR 'osteoarthritis' OR osteoarthritis:ti,ab,kw OR osteoarthritides:ti,ab,kw OR osteoarthrosis:ti,ab,kw OR osteoarthroses:ti,ab,kw OR arthrosis:ti,ab,kw OR arthroses:ti,ab,kw) AND [english]/lim	141327
**2**	'tumor necrosis factor inhibitor'/exp OR 'tumor necrosis factor inhibitor*':ti,ab,kw OR 'tnf antagonist*':ti,ab,kw OR 'tnf inhibitor*':ti,ab,kw OR 'tnf blocker*':ti,ab,kw OR 'tumor necrosis factor antagonist*':ti,ab,kw OR 'tumor necrosis factor blocker*':ti,ab,kw OR 'tumor necrosis factor-alpha inhibitor*':ti,ab,kw OR 'tumor necrosis factor-alpha blocker*':ti,ab,kw OR 'tumor necrosis factor-alpha antagonist*':ti,ab,kw	96024
**3**	'adalimumab'/exp OR adalimumab:ti,ab,kw OR humira:ti,ab,kw OR amjevita:ti,ab,kw OR cyltezo:ti,ab,kw OR 'adalimumab adbm':ti,ab,kw OR 'adalimumab atto':ti,ab,kw	34607

**4**	'infliximab'/exp OR infliximab:ti,ab,kw OR 'sb2 inflixi-mab':ti,ab,kw OR renflexis:ti,ab,kw OR inflectra:ti,ab,kw OR remicade:ti,ab,kw OR 'monoclonal antibody ca2':ti,ab,kw OR 'mab ca2':ti,ab,kw OR 'infliximab abda':ti,ab,kw OR 'infliximab dyyb':ti,ab,kw	52156
**5**	'etanercept'/exp OR etanercept:ti,ab,kw OR humira:ti,ab,kw OR 'tnr 001':ti,ab,kw OR 'tnt receptor fusion protein':ti,ab,kw OR 'tnfr-fc fusion protein':ti,ab,kw OR 'tnfr fc fusion pro-tein':ti,ab,kw OR 'tntr-fc':ti,ab,kw OR 'tnr-001':ti,ab,kw OR 'tnr001':ti,ab,kw OR 'etanercept-szzs':ti,ab,kw OR 'tnf receptor type ii-igg fusion pro-tein':ti,ab,kw OR 'tnf receptor type ii igg fusion protein':ti,ab,kw OR 'erelzi':ti,ab,kw OR 'enbrel':ti,ab,kw OR 'recombinant hu-man dimeric tnf receptor type ii-igg fusion protein':ti,ab,kw OR 'recombinant human dimeric tnf receptor type ii igg fusion pro-tein':ti,ab,kw	32874
**6**	'golimumab'/exp OR golimumab:ti,ab,kw OR 'cnto-148':ti,ab,kw OR 'cnto 148':ti,ab,kw OR simponi:ti,ab,kw	7120
**7**	#2 OR #3 OR #4 OR #5 OR #6	97397
**8**	'amg 108'/exp OR 'amg 108':ti,ab,kw OR amg108:ti,ab,kw	46
**9**	'canakinumab'/exp OR canakinumab:ti,ab,kw OR ila-ris:ti,ab,kw OR 'acz 885':ti,ab,kw OR acz885:ti,ab,kw	3226
**10**	'lutikizumab'/exp OR lutikizumab:ti,ab,kw OR 'abt 981':ti,ab,kw	39
**11**	'gevokizumab'/exp OR gevokizumabt:ti,ab,kw OR xoma052:ti,ab,kw OR 'xoma 052':ti,ab,kw	263
**12**	#8 OR #9 OR #10 OR #11	3406
**13**	'interleukin 17'/exp OR 'interleukin 17':ti,ab,kw OR interleu-kin17:ti,ab,kw OR 'ctla 8':ti,ab,kw OR 'il 17':ti,ab,kw OR ctla8:ti,ab,kw	55181

**14**	'interleukin 6'/exp OR 'interleukin 6':ti,ab,kw OR il6:ti,ab,kw OR 'il 6':ti,ab,kw OR interleukin6:ti,ab,kw OR 'b cell stimulatory factor 2':ti,ab,kw OR 'bsf 2':ti,ab,kw OR 'b cell differentiation factor 2':ti,ab,kw OR 'hybridoma growth factor*':ti,ab,kw OR 'ifn-beta 2':ti,ab,kw OR 'plasmacytoma growth factor*':ti,ab,kw OR 'mgi 2':ti,ab,kw OR 'hepatocyte stimulating factor*':ti,ab,kw OR 'myeloid differentiation inducing protein':ti,ab,kw OR 'b cell differentiation factor*':ti,ab,kw OR 'interferon beta 2':ti,ab,kw	276123
**15**	'interleukin derivative'/exp OR 'interleukin derivative':ti,ab,kw OR interleukin*:ti,ab,kw	299170
**16**	antagonist* OR inhibitor* OR blocker*	2664028
**17**	#13 OR #14 OR #15	490151
**18**	#16 AND #17	102301
**19**	'sarilumab'/exp OR sarilumab:ti,ab,kw OR 'sar-153191':ti,ab,kw OR sar153191:ti,ab,kw OR kevzara:ti,ab,kw OR 'regn-88':ti,ab,kw OR regn88:ti,ab,kw	717
**20**	'vobarilizumab'/exp OR 'alx-0061':ti,ab,kw	58
**21**	'sant7':ti,ab,kw OR 'sant-7':ti,ab,kw	18
**22**	'satralizumab'/exp OR 'satralizumab':ti,ab,kw	74
**23**	'sirukumab'/exp OR sirukumab:ti,ab,kw OR 'cnto-136':ti,ab,kw OR 'cnto 136':ti,ab,kw	315
**24**	'siltuximab'/exp OR 'siltuximab':ti,ab,kw OR cllb8:ti,ab,kw OR 'cclb8 monoclonal antibody':ti,ab,kw OR 'sylvant':ti,ab,kw OR 'cnto-328':ti,ab,kw OR 'monoclonal antibody cnto-328':ti,ab,kw OR 'cnto 328 monoclonal antibody':ti,ab,kw OR 'monoclonal antibody cnto 328':ti,ab,kw OR 'monoclonal antibody cnto328':ti,ab,kw	815
**25**	'clazakizumab'/exp OR clazakizumab:ti,ab,kw OR 'ald-518':ti,ab,kw OR ald518:ti,ab,kw OR 'bms-945429':ti,ab,kw	202

**26**	'olokizumab'/exp OR olokizumab:ti,ab,kw OR 'cdp-6038':ti,ab,kw OR cdp6038:ti,ab,kw	87
**27**	#19 OR #20 OR #21 OR #22 OR #23 OR #24 OR #25 OR #26	1897
**28**	'secukinumab'/exp OR secukinumab:ti,ab,kw OR 'cosentyx':ti,ab,kw OR ain457:ti,ab,kw OR 'ain 457':ti,ab,kw OR 'ain-457':ti,ab,kw	3830
**29**	'ixekizumab'/exp OR ixekizumab:ti,ab,kw OR 'taltz':ti,ab,kw OR ly2439821:ti,ab,kw OR 'ly-2439821':ti,ab,kw	1849
**30**	'brodalumab'/exp OR 'brodalumab':ti,ab,kw OR siliq:ti,ab,kw OR 'khk-4827':ti,ab,kw OR 'khk4827':ti,ab,kw OR 'amg-827':ti,ab,kw OR 'amg 827':ti,ab,kw	1140
**31**	#28 OR #29 OR #30	4929
**32**	'randomized controlled trial'/exp OR 'randomized controlled trial':ti,ab,it OR 'randomized':ti,ab,it OR 'randomised':ti,ab,it OR 'randomization':ti,ab,it OR 'randomisation':ti,ab,it OR rct:ti,ab,it OR 'randomly':ti,ab,it OR placebo:ti,ab,it	1511619
**33**	'observational study'/exp OR 'non experimental studies':ti,ab,kw OR 'non experimental study':ti,ab,kw OR 'nonexperimental studies':ti,ab,kw OR 'nonexperimental study':ti,ab,kw OR 'observation study':ti,ab,kw OR 'observational studies':ti,ab,kw OR 'observational study':ti,ab,kw	262151
**34**	#32 OR #33	1743673
**35**	#1 AND #34	17785
**36**	'interleukin 1 receptor blocking agent'/exp OR 'interleukin 1 receptor blocking agent':ti,ab,kw OR anakinra:ti,ab,kw OR 'il 1ra':ti,ab,kw OR 'urine il-1 inhibitor*':ti,ab,kw OR antril:ti,ab,kw OR kineret:ti,ab,kw	18428
**37**	#12 OR #36	20995
**38**	#7 OR #18 OR #27 OR #31 OR #37	207670

**39**	#35 AND #38	398

CENTRAL

No.	Query	Results
**1**	MeSH descriptor: [Osteoarthritis] explode all trees	7458
**2**	osteoarthritis:ti,ab,kw or "Osteoarthritides":ti,ab,kw or "Osteoarthrosis":ti,ab,kw or "Osteoarthroses":ti,ab,kw or "Arthrosis":ti,ab,kw or "Arthroses":ti,ab,kw	17853
**3**	MeSH descriptor: [Randomized Controlled Trial] explode all trees	118
**4**	MeSH descriptor: [Random Allocation] explode all trees	20604
**5**	"randomized":ti,ab,kw OR "randomised":ti,ab,kw OR "randomization":ti,ab,kw OR "randomisation":ti,ab,kw OR "randomly":ti,ab,kw OR "placebo":ti,ab,kw OR "trial":ti,ab,kw OR "Random Allocation":ti,ab,kw OR "Randomized Controlled Trial":ti,ab,kw OR "observational study":ti,ab,kw	1168382
**6**	MeSH descriptor: [Observational Study] explode all trees	3
**7**	#1 OR #2	17853
**8**	#3 OR #4 OR #5 OR #6	1168382
**9**	#7 AND #8	14195
**10**	MeSH descriptor: [Tumor Necrosis Factor Inhibitors] explode all trees	36
**11**	"Tumor Necrosis Factor Inhibitor*":ti,ab,kw or "TNF Antagonist*":ti,ab,kw or "TNF Inhibitor*":ti,ab,kw or "TNF Blocker*":ti,ab,kw or "Tumor Necrosis Factor Antagonist*":ti,ab,kw or "Tumor Necrosis Factor Blocker*":ti,ab,kw or "Tumor Necrosis Factor-alpha Antagonist*":ti,ab,kw or "Tumor Necrosis Factor-alpha	1018

	Inhibitor*":ti,ab,kw or "Tumor Necrosis Factor-alpha Blocker*":ti,ab,kw	
**12**	#10 OR #11	1041
**13**	#9 AND #12	9
**14**	MeSH descriptor: [Adalimumab] explode all trees	744
**15**	Adalimumab:ti,ab,kw OR "Humira":ti,ab,kw OR "Amjevita":ti,ab,kw OR "Cyltezo":ti,ab,kw OR "Adalimumabadbm":ti,ab,kw OR "Adalimumab-atto":ti,ab,kw	3004
**16**	#14 OR #15	3004
**17**	MeSH descriptor: [Infliximab] explode all trees	722
**18**	Infliximab:ti,ab,kw OR “SB2 infliximab”:ti,ab,kw OR “Renflexis”:ti,ab,kw OR “Inflectra”:ti,ab,kw OR “Remicade”:ti,ab,kw OR "Monoclonal Antibody cA2":ti,ab,kw OR "MAb cA2":ti,ab,kw OR "Infliximababda":ti,ab,kw OR "Infliximab-dyyb":ti,ab,kw	2306
**19**	#17 OR #18	2306
**20**	MeSH descriptor: [Etanercept] explode all trees	758
**21**	"Etanercept":ti,ab,kw OR "TNFR-Fc Fusion Protein":ti,ab,kw OR "TNFR Fc Fusion Protein":ti,ab,kw OR "TNR 001":ti,ab,kw OR "TNT Receptor Fusion Protein":ti,ab,kw OR "TNTR-Fc":ti,ab,kw OR "TNR-001":ti,ab,kw OR "TNR001":ti,ab,kw OR "Etanercept-szzs":ti,ab,kw OR "TNF Receptor Type II-IgG Fusion Protein":ti,ab,kw OR "TNF Receptor Type II IgG Fusion Protein":ti,ab,kw OR "Erelzi":ti,ab,kw OR "Enbrel":ti,ab,kw OR "Recombinant Human Dimeric TNF Receptor Type II-IgG Fusion Protein":ti,ab,kw OR "Recombinant Human	2187

	Dimeric TNF Receptor Type II IgG Fusion Protein":ti,ab,kw	
**22**	#20 OR #21	2187
**23**	MeSH descriptor: [Certolizumab Pegol] explode all trees	170
**24**	Certolizumab Pegol:ti,ab,kw OR “Cimzia”:ti,ab,kw OR “ CDP870 ”:ti,ab,kw OR “CDP 870”:ti,ab,kw	587
**25**	#23 OR #24	587
**26**	golimumabl:ti,ab,kw OR “CNTO-148”:ti,ab,kw OR “ CNTO 148 ”:ti,ab,kw OR “Simponi ”:ti,ab,kw	54
**27**	#12 OR #16 OR #19 OR #22 OR #25 OR #26	7375
**28**	“AMG 108”:ti,ab,kw OR “AMG-108”:ti,ab,kw OR “AMG108”:ti,ab,kw	8
**29**	"Canakinumab":ti,ab,kw OR "ilaris":ti,ab,kw OR "ACZ-885":ti,ab,kw OR "ACZ885":ti,ab,kw	308
**30**	lutikizumab:ti,ab,kw OR “ABT-981”:ti,ab,kw	8
**31**	gevokizumab:ti,ab,kw OR "gevokizumab":ti,ab,kw OR "XOMA 052":ti,ab,kw OR " XOMA052":ti,ab,kw OR "XOMA-052":ti,ab,kw	33
**32**	MeSH descriptor: [Interleukin 1 Receptor Antagonist Protein] explode all trees	307
**33**	“Interleukin 1 Receptor Antagonist Protein”:ti,ab,kw OR “Anakinra”:ti,ab,kw OR “IL-1Ra”:ti,ab,kw OR “Urine IL-1 Inhibitor”:ti,ab,kw OR “Antril”:ti,ab,kw OR “Kineret”:ti,ab,kw	519
**34**	#28 OR #29 OR #30 OR #31 OR #32 OR #33	851

**35**	"antagonists and inhibitors":ti,ab,kw OR "inhibitor*":ti,ab,kw OR "antagonist*":ti,ab,kw OR “blocker*”:ti,ab,kw	79776
**36**	MeSH descriptor: [Interleukin-17] explode all trees	167
**37**	"interleukin 17":ti,ab,kw OR "CTLA-8":ti,ab,kw OR "IL-17":ti,ab,kw OR "CTLA8":ti,ab,kw OR "interleukin-17":ti,ab,kw	1777
**38**	MeSH descriptor: [Interleukin-6] explode all trees	3022
**39**	"interleukin-6":ti,ab,kw OR "interleukin 6":ti,ab,kw OR "IL6":ti,ab,kw OR "IL-6":ti,ab,kw OR "b cell stimulatory factor 2":ti,ab,kw OR "b cell stimulatory factor-2":ti,ab,kw OR "BSF-2":ti,ab,kw OR "b cell differentiation factor 2":ti,ab,kw OR "b cell differentiation factor-2":ti,ab,kw OR "Hybridoma Growth Factor":ti,ab,kw OR "IFN-beta 2":ti,ab,kw OR "Plasmacytoma Growth Factor":ti,ab,kw OR "MGI-2":ti,ab,kw OR "hepatocyte stimulating factor":ti,ab,kw OR "hepatocyte-stimulating factor":ti,ab,kw OR "myeloid differentiation-inducing protein":ti,ab,kw OR "myeloid differentiation inducing protein":ti,ab,kw OR "b-cell differentiation factor":ti,ab,kw OR "b cell differentiation factor":ti,ab,kw OR "interferon beta 2":ti,ab,kw OR "interferon beta-2":ti,ab,kw OR "b cell stimulatory factor 2":ti,ab,kw OR "b cell stimulatory factor-2":ti,ab,kw	15947
**40**	MeSH descriptor: [Interleukins] explode all trees	6291
**41**	interleukin*:ti,ab,kw	19901
**42**	#35 AND (#36 OR #37 OR #38 OR #39 OR #40 OR #41)	2942
**43**	"sarilumab":ti,ab,kw OR "SAR-153191":ti,ab,kw OR "SAR153191":ti,ab,kw OR "Kevzara":ti,ab,kw OR "REGN-88":ti,ab,kw OR "REGN88":ti,ab,kw OR "TNR-001":ti,ab,kw OR "TNR001":ti,ab,kw OR "Etanerceptszzs":ti,ab,kw OR "TNF Receptor Type II-IgG Fusion Protein":ti,ab,kw	239

**44**	"ALX-0061":ti,ab,kw OR "vobarilizumab":ti,ab,kw	17
**45**	"SANT-7":ti,ab,kw OR "SANT7":ti,ab,kw	0
**46**	"satralizumab":ti,ab,kw	23
**47**	"sirukumab":ti,ab,kw OR "CNTO-136":ti,ab,kw OR "CNTO 136":ti,ab,kw	108
**48**	"siltuximab":ti,ab,kw OR "CLLB8":ti,ab,kw OR "cClB8 monoclonal antibody":ti,ab,kw OR "Sylvant":ti,ab,kw OR "CNTO-328":ti,ab,kw OR "monoclonal antibody CNTO-328":ti,ab,kw OR "CNTO 328 monoclonal anti-body":ti,ab,kw OR "monoclonal antibody CNTO 328":ti,ab,kw OR "monoclonal antibody CNTO328":ti,ab,kw	68
**49**	"clazakizumab":ti,ab,kw OR "ALD-518":ti,ab,kw OR "ALD518":ti,ab,kw OR "BMS-945429":ti,ab,kw	37
**50**	"olokizumab":ti,ab,kw OR "CDP-6038":ti,ab,kw OR "CDP6038":ti,ab,kw	34
**51**	"tocilizumab":ti,ab,kw OR "RHPM-1":ti,ab,kw OR "RG-1569":ti,ab,kw OR "R-1569":ti,ab,kw OR "MSB11456":ti,ab,kw OR "MSB-11456":ti,ab,kw OR "at-lizumab":ti,ab,kw OR "RO-4877533":ti,ab,kw OR "Actemra":ti,ab,kw OR "roactemra":ti,ab,kw	1073
**52**	#43 OR #44 OR #45 OR #46 OR #47 OR #48 OR #49 OR #50 OR #51	1545
**53**	"Secukinumab":ti,ab,kw OR "Cosentyx":ti,ab,kw OR "AIN 457":ti,ab,kw OR "AIN457":ti,ab,kw OR "AIN-457":ti,ab,kw	837
**54**	"Ixekizumab":ti,ab,kw OR "Taltz":ti,ab,kw OR "LY2439821":ti,ab,kw OR "LY-2439821":ti,ab,kw	421
**55**	"Brodalumab":ti,ab,kw OR "Siliq":ti,ab,kw OR "KHK-4827":ti,ab,kw OR "KHK4827":ti,ab,kw OR "AMG-827":ti,ab,kw OR "AMG 827":ti,ab,kw	165
**56**	#53 OR #54 OR #55	1397

**57**	#27 OR #34 OR #42 OR #52 OR #56	12573
**58**	#9 AND #57	124

Table S3. A hierarchy list of data extraction.

Primary outcome	Rank
Pain	(1) VAS global pain score<br>(2) Pain during activities or at test<br>(3) WOMAC pain subscale<br>(4) AUSCAN pain subscale<br>(5) Other composite pain score<br>(6) Patients' global assessment<br>(7) Physicians' global assessment
Physical function/Stiffness	(1) WOMAC physical function/stiffness subscale<br>(2) AUSCAN physical function/stiffness subscale<br>(3) Other composite physical function/stiffness subscale<br>(4) patients' global assessment<br>(5) physicians' global assessment

WOMAC, Western Ontario and McMaster Universities Osteoarthritis Index; AUSCAN, Australian/Canadian Hand Osteoarthritis Index

Table S4. Rank probability of pain.

Drug	Rank 1	Rank 2	Rank 3	Rank 4	Rank 5	Rank 6	Rank 7	Rank 8	Rank 9	Rank 10
Cana-kinumab	0.79	0.14	0.03	0.01	0.01	0.01	0	0	0	0
Naproxen	0.1	0.67	0.13	0.04	0.02	0.02	0.01	0.01	0	0
Anakinra	0.07	0.08	0.19	0.09	0.1	0.13	0.09	0.13	0.12	0
Placebo	0	0.02	0.3	0.37	0.21	0.08	0.02	0	0	0
Lutikizumab	0.02	0.04	0.17	0.23	0.22	0.18	0.08	0.05	0.02	0
Tocilizumab	0.02	0.03	0.07	0.12	0.22	0.22	0.16	0.12	0.05	0
HA	0.01	0.02	0.08	0.09	0.12	0.21	0.28	0.15	0.04	0
Adalimumab	0	0.01	0.02	0.04	0.07	0.13	0.26	0.37	0.1	0
Etanercept	0	0	0.01	0.01	0.02	0.04	0.09	0.17	0.65	0.01
Infliximab	0	0	0	0	0	0	0	0	0.01	0.98

Rank 1 is the worst option; HA, hyaluronic acid

Table S5. Rank probability of physical function.

Drug	Rank 1	Rank 2	Rank 3	Rank 4	Rank 5	Rank 6	Rank 7	Rank 8
HA	0.46	0.32	0.08	0.05	0.03	0.03	0.03	0.01
Cana-kinumab	0.08	0.22	0.26	0.16	0.09	0.07	0.07	0.05
Naproxen	0.05	0.12	0.24	0.22	0.13	0.09	0.08	0.06
Placebo	0	0.02	0.07	0.2	0.3	0.27	0.12	0.02
Adalimumab	0.01	0.04	0.12	0.13	0.16	0.19	0.21	0.15
Tocilizumab	0.04	0.06	0.08	0.1	0.13	0.18	0.22	0.18
Lutikizumab	0.03	0.06	0.09	0.11	0.12	0.14	0.21	0.24
Etanercept	0.32	0.16	0.06	0.05	0.04	0.04	0.06	0.28

Rank 1 is the worst option; HA, hyaluronic acid

Table S6. Rank probability of stiffness.

Drug	Rank 1	Rank 2	Rank 3	Rank 4	Rank 5	Rank 6	Rank 7
Etanercept	0.3	0.08	0.06	0.05	0.05	0.1	0.36
HA	0.2	0.23	0.14	0.12	0.12	0.13	0.05
Adalimumab	0.09	0.2	0.22	0.17	0.17	0.11	0.04
Placebo	0.03	0.07	0.17	0.22	0.26	0.2	0.05
Naproxen	0.11	0.17	0.16	0.18	0.17	0.14	0.08
Cana-kinumab	0.21	0.2	0.18	0.16	0.12	0.08	0.05
Lutikizumab	0.06	0.06	0.07	0.1	0.11	0.24	0.37

Rank 1 is the worst option; HA, hyaluronic acid

Table S7. Network meta-analysis of stiffness for different interventions.

**HA**	**-0.20 (-3.20, 2.81)**	**-0.03 (-5.47, 5.19)**	**-0.74 (-7.43, 5.63)**	**-1.40 (-7.00, 4.02)**	**-0.33 (-5.81, 4.91)**	**-0.60 (-5.07, 3.67)**
0.20 (-2.81, 3.20)	**adalimumab**	0.17 (-4.19, 4.48)	-0.55 (-7.70, 6.32)	-1.20 (-5.81, 3.21)	-0.14 (-4.52, 4.08)	-0.40 (-3.59, 2.58)
0.03 (-5.19, 5.47)	-0.17 (-4.48, 4.19)	**canakinumab**	-0.70 (-8.95, 7.42)	-1.39 (-5.93, 3.14)	-0.30 (-3.39, 2.69)	-0.57 (-3.65, 2.39)
0.74 (-5.63, 7.43)	0.55 (-6.32, 7.70)	0.70 (-7.42, 8.95)	**etanercept**	-0.68 (-8.75, 7.71)	0.39 (-7.83, 8.77)	0.12 (-7.44, 7.92)
1.40 (-4.02, 7.00)	1.20 (-3.21, 5.81)	1.39 (-3.14, 5.93)	0.68 (-7.71, 8.75)	**lutikizumab**	1.10 (-3.44, 5.59)	0.81 (-2.54, 4.15)
0.33 (-4.91, 5.81)	0.14 (-4.08, 4.52)	0.30 (-2.69, 3.39)	-0.39 (-8.77, 7.83)	-1.10 (-5.59, 3.44)	**naproxen**	-0.29 (-3.33, 2.89)
0.60 (-3.67, 5.07)	0.40 (-2.58, 3.59)	0.57 (-2.39, 3.65)	-0.12 (-7.92, 7.44)	-0.81 (-4.15, 2.54)	0.29 (-2.89, 3.33)	**placebo**

HA, hyaluronic acid.

Table S8. Network meta-analysis of AEs for different interventions.

**AMG108**	0.94 (0.24, 3.80)	0.67 (0.13, 3.29)	0.42 (0.08, 2.13)	1.27 (0.24, 7.01)	3.44 (0.19, 152.96)	0.82 (0.19, 3.62)	0.67 (0.13, 3.50)	0.88 (0.28, 2.88)	3.42 (0.11, 223.08)	1.70 (0.30, 9.66)
1.07 (0.26, 4.24)	**adalimumab**	0.72 (0.18, 2.59)	0.45 (0.11, 1.75)	1.34 (0.32, 5.87)	3.82 (0.24, 141.19)	0.87 (0.28, 2.78)	0.71 (0.17, 2.92)	0.94 (0.43, 1.97)	3.58 (0.14, 200.33)	1.82 (0.41, 7.65)
1.49 (0.30, 7.46)	1.39 (0.39, 5.56)	**anakinra**	0.62 (0.13, 3.06)	1.87 (0.37, 9.99)	5.12 (0.28, 209.25)	1.21 (0.32, 4.96)	1.00 (0.19, 5.03)	1.30 (0.44, 4.10)	5.03 (0.17, 300.73)	2.50 (0.50, 14.16)
2.38 (0.47, 11.94)	2.22 (0.57, 8.99)	1.62 (0.33, 7.81)	**Cana-kinumab**	3.02 (0.55, 16.45)	8.18 (0.49, 345.91)	1.93 (0.49, 7.89)	1.59 (0.50, 4.88)	2.10 (0.68, 6.67)	8.00 (0.26, 440.09)	4.02 (0.73, 21.70)
0.79 (0.14, 4.22)	0.75 (0.17, 3.12)	0.53 (0.10, 2.68)	0.33 (0.06, 1.82)	**etanercept**	2.83 (0.14, 117.56)	0.64 (0.15, 3.03)	0.52 (0.09, 2.78)	0.69 (0.20, 2.33)	2.57 (0.10, 166.03)	1.37 (0.22, 7.75)
0.29 (0.01, 5.18)	0.26 (0.01, 4.14)	0.20 (0.00, 3.53)	0.12 (0.00, 2.03)	0.35 (0.01, 6.95)	**infliximab**	0.22 (0.01, 3.85)	0.19 (0.00, 3.37)	0.25 (0.01, 3.70)	0.94 (0.07, 17.66)	0.47 (0.01, 9.62)
1.23 (0.28, 5.38)	1.15 (0.36, 3.63)	0.82 (0.20, 3.15)	0.52 (0.13, 2.06)	1.56 (0.33, 6.72)	4.45 (0.26, 157.45)	**lutikizumab**	0.83 (0.19, 3.46)	1.08 (0.47, 2.55)	4.13 (0.14, 236.51)	2.09 (0.44, 9.17)
1.49 (0.29, 7.85)	1.40 (0.34, 5.88)	1.00 (0.20, 5.38)	0.63 (0.21, 2.01)	1.91 (0.36, 11.01)	5.18 (0.30, 209.79)	1.20 (0.29, 5.29)	**naproxen**	1.31 (0.40, 4.50)	5.03 (0.18, 299.50)	2.55 (0.45, 14.00)
1.13 (0.35, 3.63)	1.07 (0.51, 2.30)	0.77 (0.24, 2.26)	0.48 (0.15, 1.48)	1.44 (0.43, 5.07)	4.08 (0.27, 141.60)	0.93 (0.39, 2.14)	0.76 (0.22, 2.48)	**placebo**	3.74 (0.16, 206.90)	1.95 (0.54, 6.60)
0.29 (0.00, 8.94)	0.28 (0.00, 7.17)	0.20 (0.00, 5.82)	0.13 (0.00, 3.90)	0.39 (0.01, 9.88)	1.06 (0.06, 13.68)	0.24 (0.00, 7.01)	0.20 (0.00, 5.59)	0.27 (0.00, 6.31)	**standard care**	0.52 (0.01, 16.39)
0.59 (0.10, 3.30)	0.55 (0.13, 2.44)	0.40 (0.07, 2.01)	0.25 (0.05, 1.36)	0.73 (0.13, 4.54)	2.12 (0.10, 84.43)	0.48 (0.11, 2.29)	0.39 (0.07, 2.24)	0.51 (0.15, 1.84)	1.93 (0.06, 133.62)	**tocilizumab**

HA, hyaluronic acid; AEs, adverse events.

Table S9. Sensitivity analysis in pain, stiffness and function.

Outcome	Intervention		Std. Mean Difference (95% CI)		I^2	*P* value
			Fixed model	Random model		
Pain	Compared with placebo	Adalimumab	-0.16 (-0.42, 0.11)	-0.16 (-0.42, 0.11)	0%	0.77
		Lutikizumab	-0.08 (-0.29, 0.12)	-0.08 (-0.29, 0.14)	9%	0.29
		Canakinumab	**1.69 (1.20, 2.19)**	**1.69 (1.20, 2.19)**	NA	NA
		Anakinra	-0.04 (-0.35, 0.26)	-0.04 (-0.35, 0.26)	NA	NA
		Etanercept	**-0.47 (-0.89, -0.05)**	**-0.47 (-0.89, -0.05)**	NA	NA
		Infliximab	**-2.04 (-2.56, -1.52)**	**-2.04 (-2.56, -1.52)**	NA	NA
		Tocilizumab	**-0.6 (-1.05, -0.15)**	**-0.6 (-1.05, -0.15)**	NA	NA
	Compared with HA	Adalimumab	**-0.62 (-1.16, -0.09)**	**-0.62 (-1.16, -0.09)**	NA	NA
		Etanercept	-0.21 (-0.84, 0.42)	-0.21 (-0.84, 0.42)	NA	NA
	Compared with naproxen	Canakinumab	**0.66 (0.24, 1.08)**	**0.66 (0.24, 1.08)**	NA	NA
Function	Compared with placebo	Adalimumab	-0.06 (-0.33, 0.20)	-0.06 (-0.33, 0.20)	0%	0.99
		Lutikizumab	-0.06 (-0.27, 0.14)	-0.06 (-0.27, 0.14)	0%	0.33
		Canakinumab	**1.55 (1.07, 2.03)**	**1.55 (1.07, 2.03)**	NA	NA
		Tocilizumab	**-1.48 (-2.00, -0.97)**	**-1.48 (-2.00, -0.97)**	NA	NA
	Compared with HA	Adalimumab	**-0.88 (-1.44, -0.33)**	**-0.88 (-1.44, -0.33)**	NA	NA
		Etanercept	-0.09 (-0.72, 0.53)	-0.09 (-0.72, 0.53)	NA	NA
	Compared with naproxen	Canakinumab	**0.52 (0.10, 0.94)**	**0.52 (0.10, 0.94)**	NA	NA
Stiffness	Compared with placebo	Adalimumab	0.10 (-0.23, 0.43)	0.10 (-0.23, 0.43)	0%	0.95
		Lutikizumab	-0.20 (-0.57, 0.18)	-0.20 (-0.57, 0.18)	NA	NA
		Canakinumab	**1.61 (1.12, 2.09)**	**1.61 (1.12, 2.09)**	NA	NA
	Compared with HA	Adalimumab	-0.14 (-0.66, 0.39)	-0.14 (-0.66, 0.39)	NA	NA
		Etanercept	-0.09 (-0.72, 0.54)	-0.09 (-0.72, 0.54)	NA	NA
	Compared with naproxen	Canakinumab	**0.83 (0.40, 1.25)**	**0.83 (0.40, 1.25)**	NA	NA

HA, hyaluronic acid; NA, not applicable

Table S10. Sensitivity analysis in adverse events.

Intervention		Odds Ratio (95% CI)		I^2	*P* value
		Fixed model	Random model		
Compared with placebo	Adalimumab	1.04 (0.59, 1.85)	1.07 (0.51, 2.26)	39%	0.20
	Etanercept	1.44 (0.62, 3.34)	1.44 (0.62, 3.34)	NA	NA
	Lutikizumab	0.92 (0.49, 1.72)	0.93 (0.49, 1.74)	0%	0.75
	Anakinra	0.75 (0.41, 1.40)	0.75 (0.41, 1.40)	NA	NA
	Canakinumab	0.48 (0.23, 1.01)	0.48 (0.23, 1.01)	NA	NA
	Infliximab	3.00 (0.21, 42.62)	3.00 (0.21, 42.62)	NA	NA
	AMG108	1.13 (0.53, 2.43)	1.13 (0.53, 2.43)	NA	NA
	Tocilizumab	1.93 (0.79, 4.72)	1.93 (0.79, 4.72)	NA	NA
Compared with HA	Etanercept	NA	NA	NA	NA
	Adalimumab	3.11 (0.12, 79.64)	3.11 (0.12, 79.64)	NA	NA
Compared with naproxen	Canakinumab	0.64 (0.31, 1.32)	0.64 (0.31, 1.32)	NA	NA
Compared with standard care	Infliximab	1.00 (0.09, 11.03)	1.00 (0.09, 11.03)	NA	NA

HA, hyaluronic acid; NA, not applicable

Table S11. Node-splitting analyses of pain.

Name	Direct Effect	Indirect Effect	Overall	P-Value
HA, adalimumab	-1.69 (-12.25, 9.85)	4.54 (-14.43, 25.30)	-1.29 (-7.19, 6.82)	0.44
HA, etanercept	-3.30 (-15.23, 10.66)	-9.88 (-28.73, 10.51)	-4.34 (-12.06, 3.83)	0.46
Adalimumab, placebo	3.29 (-5.19, 10.98)	9.59 (-11.05, 32.53)	4.05 (-1.85, 9.67)	0.47
Etanercept, placebo	9.91 (-2.99, 22.59)	3.34 (-14.51, 20.24)	7.49 (-0.57, 15.93)	0.44

HA, hyaluronic acid

Table S12. ISD of inconsistency test.

Outcome	Inconsistency standard deviation [median(95% CI)]
Pain	8.82 (0.25, 24.50)
Physical function	8.21 (0.39, 15.91)
Stiffness	1.20 (0.06, 2.34)
AEs	**0.43 (0.03, 0.82)**

ISD, Inconsistency Standard Deviation; AEs, adverse events

Table S13. Results of Egger's test.

Outcome	Std Eff	Coef.	Std. Err	t	P > |t|	95% Conf. Interval
Pain	Slope	-0.06	0.85	-0.07	0.943	[-1.93, 1.80]
	bias	0.11	4.11	0.03	0.980	[-8.94, 9.16]
Function	Slope	-0.56	0.75	-0.75	0.474	[-2.29, 1.16]
	bias	3.68	3.58	1.03	0.335	[-4.59, 11.95]
Stiffness	Slope	-0.367	2.01	-0.18	0.861	[-5.27, 4.54]
	bias	2.43	8.60	0.28	0.787	[-18.62, 23.49]
AEs	Slope	-0.38	0.33	-1.13	0.277	[-1.09, 0.34]
	bias	0.85	0.69	1.22	0.242	[-0.64, 2.34]

AEs, adverse events

Study or Subgroup	Experimental Mean	Experimental SD	Experimental Total	Control Mean	Control SD	Control Total	Weight	Std. Mean Difference IV, Fixed, 95% CI
**1.1.1 adalimumab vs placebo**								
aitken2018	−3.2	16.7	41	−0.8	29.6	39	37.2%	−0.10 [−0.54, 0.34]
chevalier2014	−19.3	26.23	39	−16.8	26.16	37	35.3%	−0.09 [−0.54, 0.36]
verbruggen2014	−5.4	9.8	30	−1.7	13.1	30	27.6%	−0.32 [−0.83, 0.19]
**Subtotal (95% CI)**			**110**			**106**	**100.0%**	**−0.16 [−0.42, 0.11]**
Heterogeneity: Chi² = 0.51, df = 2 (P = 0.77); I² = 0%								
Test for overall effect: Z = 1.15 (P = 0.25)								
**1.1.2 lutikizumab vs placebo**								
fleischmann2019	−10.35	9.43	262	−8.9	9.5	85	69.8%	−0.15 [−0.40, 0.09]
kloppenbrug2018	−9.2	16.36	51	−10.7	18.35	61	30.2%	0.09 [−0.29, 0.46]
**Subtotal (95% CI)**			**313**			**146**	**100.0%**	**−0.08 [−0.29, 0.12]**
Heterogeneity: Chi² = 1.10, df = 1 (P = 0.29); I² = 9%								
Test for overall effect: Z = 0.78 (P = 0.44)								
**1.1.3 canakinumab vs placebo**								
NCT01160822	−25.1	4.14	43	−32.1	4.06	44	100.0%	1.69 [1.20, 2.19]
**Subtotal (95% CI)**			**43**			**44**	**100.0%**	**1.69 [1.20, 2.19]**
Heterogeneity: Not applicable								
Test for overall effect: Z = 6.73 (P < 0.00001)								
**1.1.4 anakinra vs placebo**								
chevalier2009	−24.8	29.11	101	−23.6	26.9	69	100.0%	−0.04 [−0.35, 0.26]
**Subtotal (95% CI)**			**101**			**69**	**100.0%**	**−0.04 [−0.35, 0.26]**
Heterogeneity: Not applicable								
Test for overall effect: Z = 0.27 (P = 0.79)								
**1.1.5 etanercept vs placebo**								
kloppenbrug2018a	−31.9	21.65	45	−21.9	20.19	45	100.0%	−0.47 [−0.89, −0.05]
**Subtotal (95% CI)**			**45**			**45**	**100.0%**	**−0.47 [−0.89, −0.05]**
Heterogeneity: Not applicable								
Test for overall effect: Z = 2.21 (P = 0.03)								
**1.1.6 infliximab vs placebo**								
fioravanti2009	−23.17	11.64	56	2.36	13.58	34	100.0%	−2.04 [−2.56, −1.52]
**Subtotal (95% CI)**			**56**			**34**	**100.0%**	**−2.04 [−2.56, −1.52]**
Heterogeneity: Not applicable								
Test for overall effect: Z = 7.63 (P < 0.00001)								
**1.1.7 canakinumab vs naproxen**								
NCT01160822	−25.1	4.14	43	−27.8	3.94	49	100.0%	0.66 [0.24, 1.08]
**Subtotal (95% CI)**			**43**			**49**	**100.0%**	**0.66 [0.24, 1.08]**
Heterogeneity: Not applicable								
Test for overall effect: Z = 3.09 (P = 0.002)								
**1.1.8 adalimumab vs HA**								
wang j2017	−3	3.35	28	−1.3	1.81	28	100.0%	−0.62 [−1.16, −0.09]
**Subtotal (95% CI)**			**28**			**28**	**100.0%**	**−0.62 [−1.16, −0.09]**
Heterogeneity: Not applicable								
Test for overall effect: Z = 2.27 (P = 0.02)								
**1.1.9 etanercept vs HA**								
ohtori2015	−2.7	9.44	19	−0.4	11.91	20	100.0%	−0.21 [−0.84, 0.42]
**Subtotal (95% CI)**			**19**			**20**	**100.0%**	**−0.21 [−0.84, 0.42]**
Heterogeneity: Not applicable								
Test for overall effect: Z = 0.65 (P = 0.52)								
**1.1.10 tocilizumab vs placebo**								
Pascal Richette2020	−13.5	3.1	41	−11.6	3.2	38	100.0%	−0.60 [−1.05, −0.15]
**Subtotal (95% CI)**			**41**			**38**	**100.0%**	**−0.60 [−1.05, −0.15]**
Heterogeneity: Not applicable								
Test for overall effect: Z = 2.59 (P = 0.009)								

Test for subgroup differences: Chi² = 127.80, df = 9 (P < 0.00001), I² = 93.0%

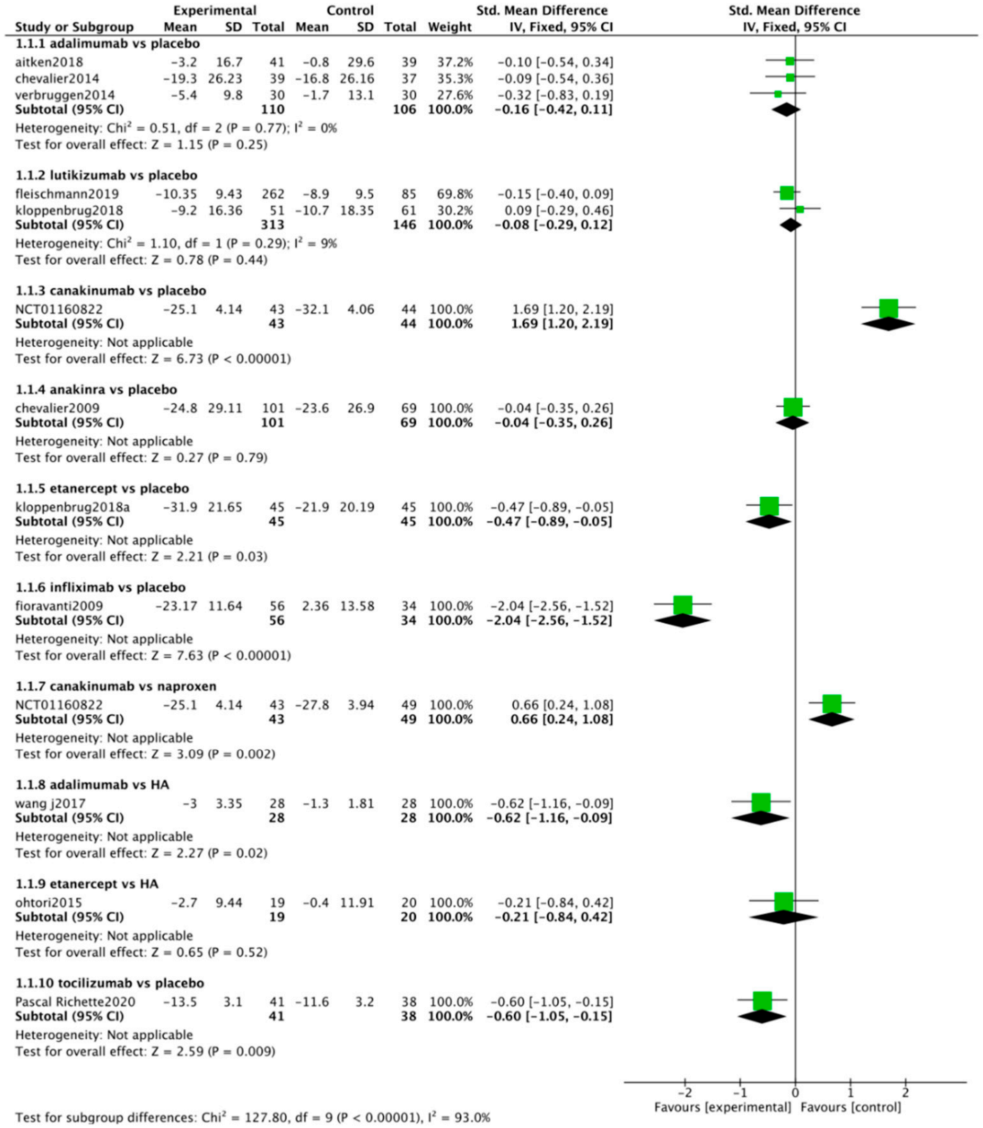

Figure S1. Results of the conventional meta-analysis of pain. HA: hyalutonic acid.

Study or Subgroup	Experimental Mean	Experimental SD	Experimental Total	Control Mean	Control SD	Control Total	Weight	Std. Mean Difference IV, Fixed, 95% CI
**2.1.1 adalimumab vs placebo**								
aitken2018	−23	136.9	41	−6.7	215.8	40	37.2%	−0.09 [−0.53, 0.35]
chevalier2014	−4	14.55	40	−3.2	14.6	37	35.3%	−0.05 [−0.50, 0.39]
verbruggen2014	1.2	18.5	30	2	17.9	30	27.5%	−0.04 [−0.55, 0.46]
**Subtotal (95% CI)**			**111**			**107**	**100.0%**	**−0.06 [−0.33, 0.20]**
Heterogeneity: $Chi^2 = 0.02$, df = 2 (P = 0.99); $I^2 = 0\%$								
Test for overall effect: Z = 0.48 (P = 0.63)								
**2.1.2 lutikizumab vs placebo**								
fleischmann2019	−32.68	30.72	262	−28.7	30.37	85	69.8%	−0.13 [−0.37, 0.12]
kloppenbrug2018	−14.6	26.67	51	−17.2	30.26	61	30.2%	0.09 [−0.28, 0.46]
**Subtotal (95% CI)**			**313**			**146**	**100.0%**	**−0.06 [−0.27, 0.14]**
Heterogeneity: $Chi^2 = 0.93$, df = 1 (P = 0.33); $I^2 = 0\%$								
Test for overall effect: Z = 0.61 (P = 0.54)								
**2.1.3 canakinumab vs placebo**								
NCT01160822	−13.4	2.01	43	−16.5	1.96	44	100.0%	1.55 [1.07, 2.03]
**Subtotal (95% CI)**			**43**			**44**	**100.0%**	**1.55 [1.07, 2.03]**
Heterogeneity: Not applicable								
Test for overall effect: Z = 6.30 (P < 0.00001)								
**2.1.4 adalimumab vs HA**								
wang j2017	−17.7	15.86	28	−6.4	8.12	28	100.0%	−0.88 [−1.44, −0.33]
**Subtotal (95% CI)**			**28**			**28**	**100.0%**	**−0.88 [−1.44, −0.33]**
Heterogeneity: Not applicable								
Test for overall effect: Z = 3.15 (P = 0.002)								
**2.1.5 canakinumab vs naproxen**								
NCT01160822	−13.4	2.01	43	−14.4	1.8	49	100.0%	0.52 [0.10, 0.94]
**Subtotal (95% CI)**			**43**			**49**	**100.0%**	**0.52 [0.10, 0.94]**
Heterogeneity: Not applicable								
Test for overall effect: Z = 2.45 (P = 0.01)								
**2.1.6 etanercept vs HA**								
ohtori2015	−12.7	43.53	19	−8.4	46.56	20	100.0%	−0.09 [−0.72, 0.53]
**Subtotal (95% CI)**			**19**			**20**	**100.0%**	**−0.09 [−0.72, 0.53]**
Heterogeneity: Not applicable								
Test for overall effect: Z = 0.29 (P = 0.77)								
**2.1.7 tocilizumab vs placebo**								
Pascal Richette2020	−1	0.6	39	−0.1	0.6	35	100.0%	−1.48 [−2.00, −0.97]
**Subtotal (95% CI)**			**39**			**35**	**100.0%**	**−1.48 [−2.00, −0.97]**
Heterogeneity: Not applicable								
Test for overall effect: Z = 5.61 (P < 0.00001)								

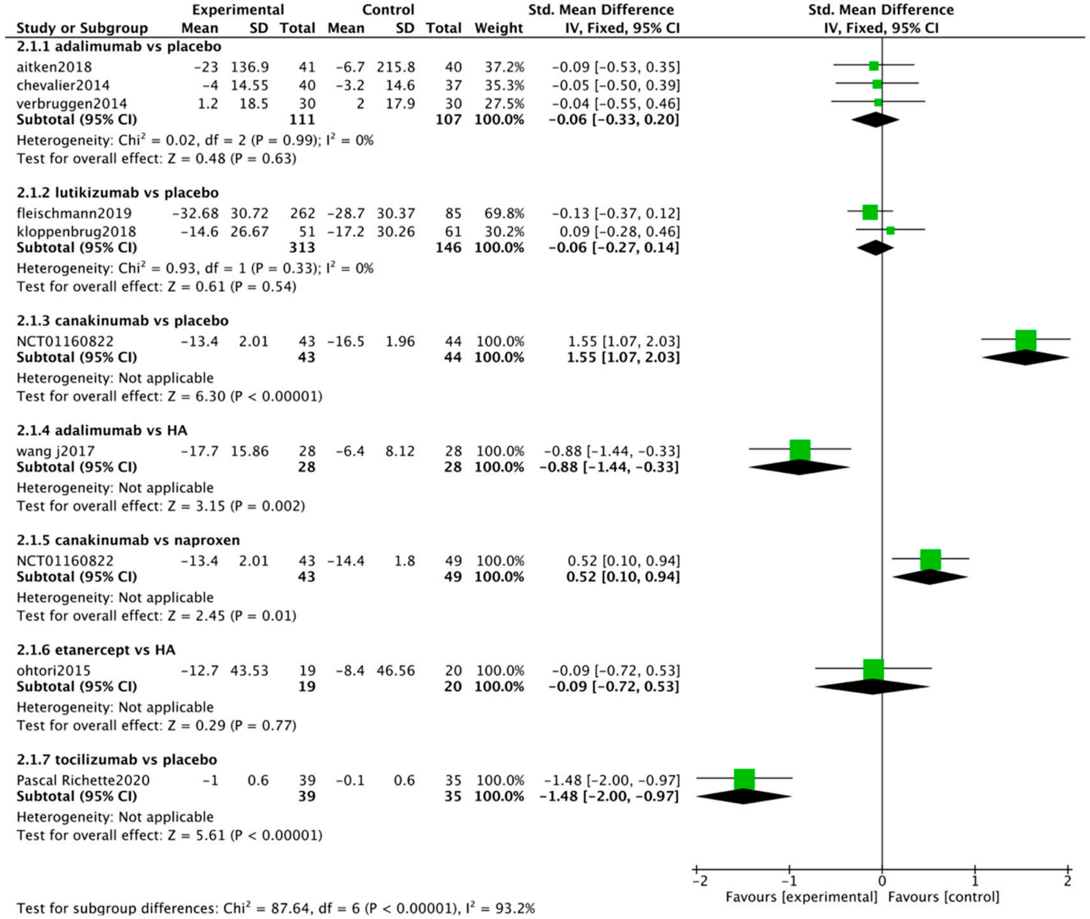

Test for subgroup differences: $Chi^2 = 87.64$, df = 6 (P < 0.00001), $I^2 = 93.2\%$

Figure S2. Results of the conventional meta-analysis of physical function. HA: hyalutonic acid.

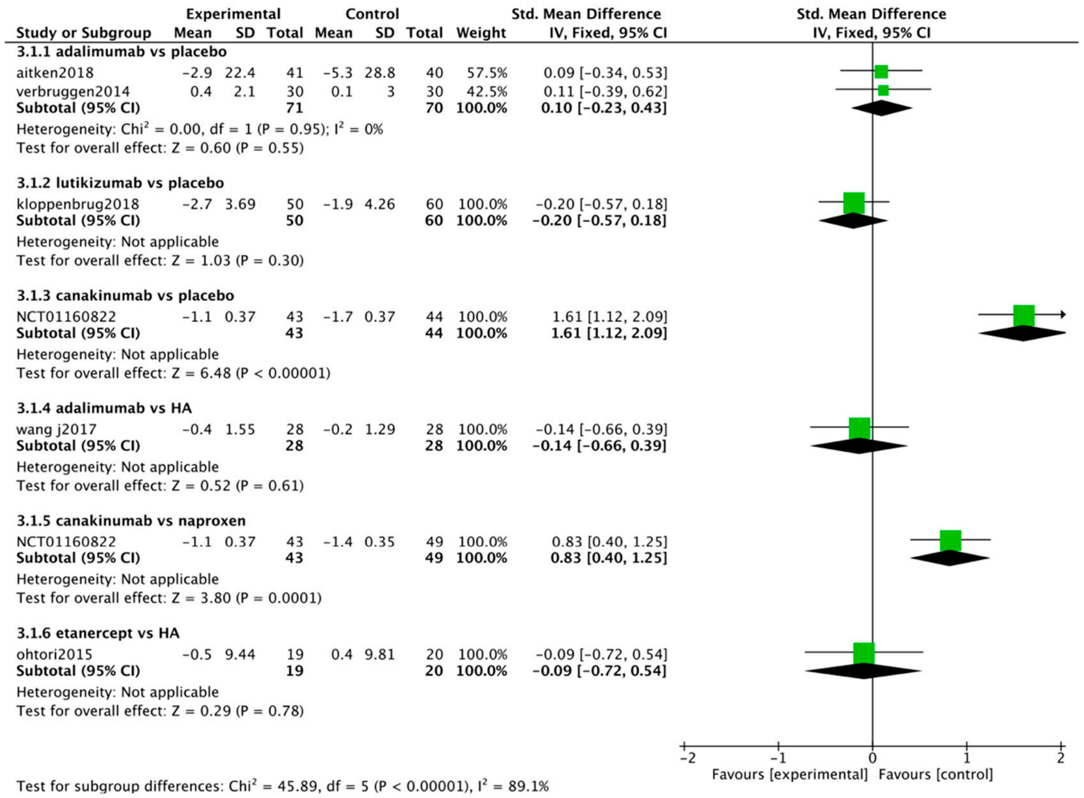

Figure S3. Results of the conventional meta-analysis of stiffness. HA: hyalutonic acid.

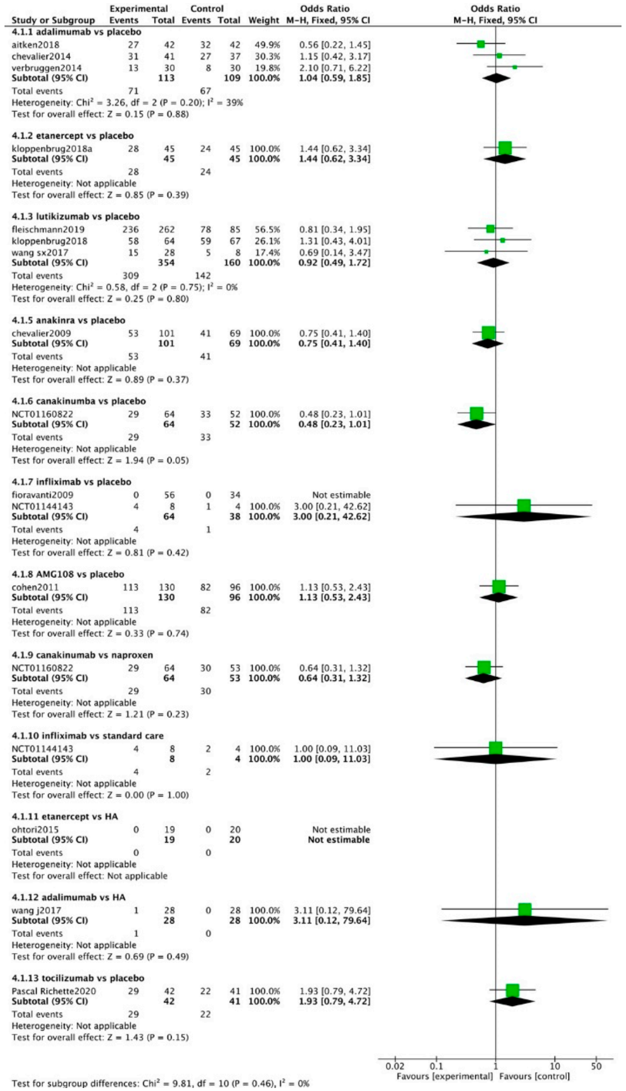

Figure S4. Results of the conventional meta-analysis of adverse events. HA: hyalutonic acid.

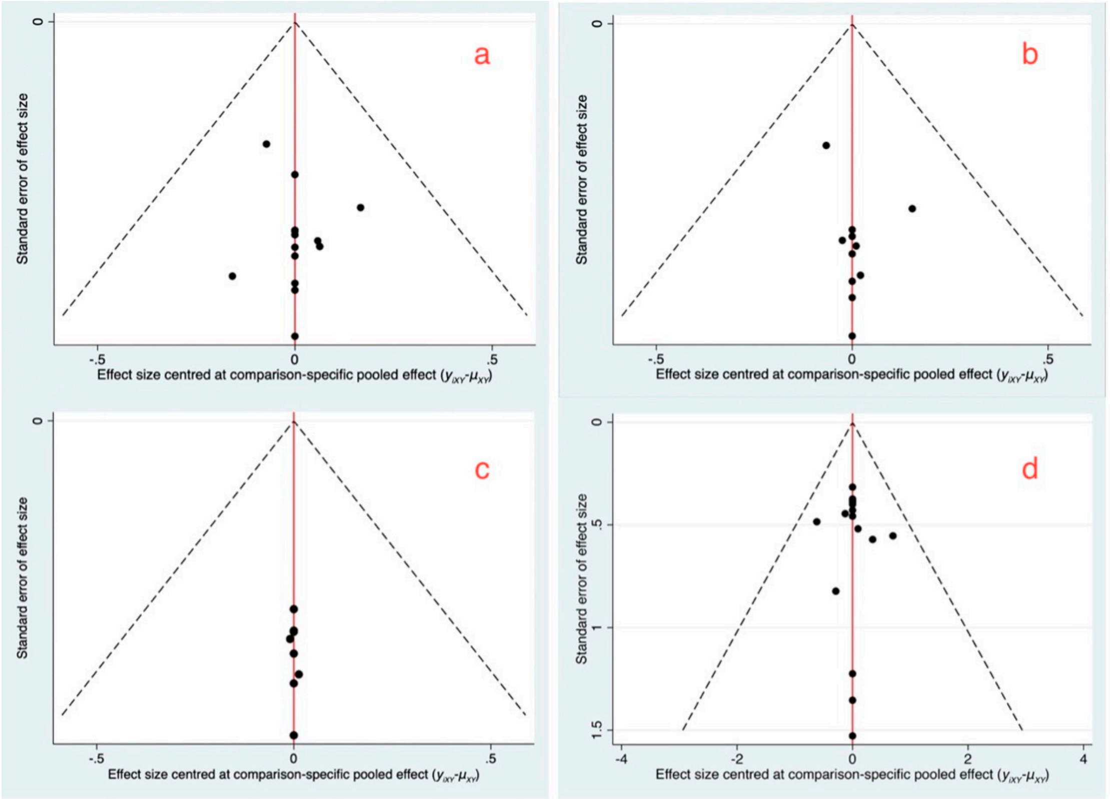

Figure S5. Publication bias examined by funnel plot. a: pain; b: physical function; c: stiffness; d: adverse events.
